# EfficientNetB5-Based Deep Learning for Automated Cancer Detection in Tissue Microarray Images

**DOI:** 10.7150/jca.139302

**Published:** 2026-09-24

**Authors:** Kuo-Wang Tsai, Bach-Tung Pham, Ching-Feng Cheng, Chun-Fan Lung, Wenny Ramadha Putri, Farchan Raswa, Jia-Ching Wang, Yi-Chiung Hsu

**Affiliations:** 1 Department of Research, Taipei Tzu Chi Hospital, Buddhist Tzu Chi Medical Foundation, New Taipei, Taiwan.; 2 Department of Nursing, Cardinal Tien Junior College of Healthcare and Management, Taiwan.; 3 Department of Biomedical Sciences and Engineering, National Central University, Taoyuan, Taiwan.; 4 Department of Computer Science and Information Engineering, National Central University, Taoyuan 320, Taiwan.; 5 Department of Pediatrics, Taipei Tzu Chi Hospital, Buddhist Tzu Chi Medical Foundation, Taipei, Taiwan.; 6 Institute of Biomedical Sciences, Academia Sinica, Taipei, Taiwan.; 7 Department of Pediatrics, Tzu Chi University, Hualien, Taiwan.; 8 Center for Astronautical Physics and Engineering, National Central University, Taoyuan City, Taiwan.; 9 Department of Medical Research, Cathay General Hospital, Taipei, Taiwan.

**Keywords:** tissue microarray, deep learning, efficientnetb5, cancer detection, histopathology image analysis, artificial intelligence

## Abstract

**Background:**

Tissue microarray (TMA) technology is pivotal in cancer research, enabling simultaneous analysis of multiple samples on a single slide. Manual analysis of TMA images remains time-consuming and subjective. This study aimed to develop a robust deep learning model to automate TMA image analysis, enhance accuracy, and support cancer diagnosis.

**Methods:**

We utilized the EfficientNetB5 deep learning architecture to classify TMA images from the DigitalSlide01 v1_1M01 dataset, comprising breast cancer, Upper Tract Urothelial Carcinoma (UTUC), and gastric cancer samples. Data augmentation methods, including Mix-Up and Cut-Mix, were employed to improve generalization. Preprocessing involved Otsu thresholding and smart bounding box cropping. The model was trained over 80 epochs using the Adam optimizer, a learning rate scheduler, and categorical cross-entropy loss. Performance was evaluated using 10-fold cross-validation and a separate test set, with accuracy, precision, recall, and F1-score as the primary metrics. Performance was further examined using physical-array-held-out and sample-level analyses.

**Results:**

Across the 10 cross-validation validation folds, mean accuracy and F1-score were 85.93% and 82.78%, respectively. Across the separate test-set evaluations by the ten fold-specific models, mean accuracy and F1-score were 80.08% and 75.13%, respectively. In the sample-level analysis, a 10-model mean-probability ensemble achieved 87.80% accuracy and 78.18% macro F1-score on the 246-core test subset. Cancer precision was 100.00% in the reported ensemble analysis, indicating that no Normal or Others samples were observed among samples predicted as Cancer; this does not imply that all Cancer samples were detected.

**Conclusions:**

The EfficientNetB5-based framework showed useful aggregate classification performance. These findings support the feasibility of automated TMA image classification and emphasize the need for source-balanced external multicenter validation before broader clinical application.

## Introduction

Cancer represents a significant global health challenge, responsible for millions of deaths annually. In efforts to uncover the underlying mechanisms of cancer development and advance treatment strategies, extensive analysis of tissue samples has become a research priority. Tissue microarray (TMA) technology has emerged as a powerful tool in cancer research, enabling the simultaneous examination of multiple samples on a single slide, thereby enhancing efficiency and comparability in this domain 1. The application of deep learning methods for automating TMA image analysis has gained considerable interest 2. In a study published in Scientific Reports, Nguyen et al. employed an ensemble deep learning approach to classify colorectal cancer tissue images. Their dataset included hematoxylin and eosin (H&E)-stained core images, extracted from 54 digital slides comprising a total of 15,150 cores from three international cohorts. Following extraction and color enhancement, five different independent and ensemble deep learning pipelines were applied to the TMA cores. The best-performing approach, a Soft Voting Ensemble combining one VGG (Visual Geometry Group) and one CapsNet (Capsule Network) model, achieved remarkable prediction accuracies, surpassing both independent and ensemble learning approaches with a single base estimator 3. In addition, Janowczyk and Madabhushi demonstrated the application of deep learning to multiple digital pathology tasks, including detection, segmentation, and tissue classification, highlighting the utility of learned image representations for histopathological analysis 4. Furthermore, Jiang et al. investigated convolutional neural networks (CNNs) for automated breast cancer histopathology image classification. Their model classified BreakHis images into benign and malignant categories and eight histological subtypes, achieving high classification accuracy and underscoring the potential of deep learning techniques in automated cancer diagnosis 5. In another significant study, Linkon et al. explored the utilization of deep learning for identifying prostate cancer in histopathology images. They developed a deep neural network model capable of classifying tissue patches as cancerous or non-cancerous. This model exhibited excellent performance in distinguishing between normal and cancerous prostate tissues, further illustrating the potential of deep learning to assist pathologists in achieving accurate cancer diagnoses 6. These studies collectively highlight the growing interest in and success of employing deep learning methods for automating the analysis of TMA images. By leveraging deep neural networks and learning image representations, researchers have made substantial advancements in the accurate classification of various cancer types. These findings emphasize the significance of developing automated systems to aid pathologists and researchers in analyzing large-scale tissue samples, ultimately contributing to enhanced cancer diagnosis and treatment. Despite advancements in deep learning methods for automating TMA image analysis, several limitations and challenges have emerged in the existing literature. Firstly, there is a notable scarcity of annotated and well-curated histopathology datasets that encompass a diverse range of cancer types and histopathological variations 7. This lack of comprehensive and standardized datasets hinders the development and evaluation of robust deep learning models for TMA image analysis. Additionally, the interpretability of deep learning models within the context of TMA analysis is often insufficient, making it challenging for researchers and clinicians to understand the underlying features and decision-making processes of these models 8, 9. Moreover, the generalizability of deep learning models across different TMA image acquisition platforms, staining protocols, and tissue preparation techniques poses a considerable challenge 10, 11. Variations in staining and scanner color response can affect model performance 12, 13, while acquisition-related domain shifts across sites or vendor platforms may further compromise reliability 14, 15. Motivated by the limitations and challenges identified in previous research, this study aims to address the need for accurate and automated TMA image analysis in cancer research. By leveraging deep learning methodologies, we seek to develop an automated system for classifying TMA images from the DigitalSlide01 v1_1M01 study dataset, which was assembled from the breast cancer, Upper Tract Urothelial Carcinoma (UTUC), and gastric cancer TMA cohorts.

## Materials and Methods

### Data Preparation and Procedure

A total of 568 specimens were included in this research, spanning gastric cancer, breast cancer, and UTUC (Table 1). These paraffin-embedded samples were procured from the Department of Pathology at Taipei Tzu Chi Hospital in Taiwan. This study was approved by the Institutional Review Board of Taipei Tzu Chi Hospital (approval numbers: 11-XD-133). The requirement for informed consent was waived due to the retrospective nature of the study and the use of de-identified archived specimens. All procedures were conducted in accordance with the Declaration of Helsinki. As the study utilized pre-existing, de-identified data and specimens, the requirement for written informed consent was waived by the hospital's IRB. This investigation utilized three distinct tissue TMA cohorts assembled from relevant studies. These cohorts consisted of paraffin-embedded samples derived from breast cancer (comprising 40 samples of normal breast tissue, 309 corresponding adjacent normal tissues, 309 cases of ductal carcinoma in situ, and 309 cases of invasive ductal carcinoma), gastric cancer (comprising 110 corresponding adjacent normal tissues and 110 gastric tumor samples), and UTUC (comprising 149 corresponding adjacent normal tissues and 149 UTUC tumor samples). Each cancer type was represented by three blocks, featuring both the respective adjacent normal tissue and tumor samples in each specimen. These specimens were obtained from 309 breast cancer patients, 110 gastric cancer patients, and 149 UTUC patients. To establish the TMA, a seasoned pathologist first identified representative tumor areas and corresponding adjacent normal tissues from hematoxylin-eosin-stained sections. These areas were then used to extract cylindrical tissue cores from paraffin-embedded samples. Subsequently, one core was extracted for each corresponding adjacent normal tissue type, while two cores were taken from tumor tissues. After creating the TMA blocks, each block was carefully sectioned into 6-µm-thick paraffin sections using a microtome and established techniques. They were deparaffinized using xylene and gradually rehydrated by immersing them in sequentially decreasing concentrations of ethanol, culminating in placement in deionized water. This process prepared the sections for subsequent H&E staining. For hematoxylin staining, a hematoxylin solution (Merck, CA, USA) was applied for 3 minutes, followed by a 5-minute water wash. Eosin staining (Merck, CA, USA) was performed for 30 seconds, and the sections were then dehydrated using incremental ethanol concentrations. The finalized sections were preserved in xylene and affixed with Micromount (Leica, CA, USA) at room temperature, allowing for mounting over a period of 1 hour.

### Datasets

The image classification task comprised three categories: Normal, Cancer, and Others. The Others class included the original Recurrent and Tumor categories, which were combined according to the predefined label-harmonization scheme used for the three-class classification task. Consistent with the sampling design described above, each cancer cohort included corresponding adjacent normal and tumor tissues. The image dataset comprised multiple physical slide/array sources representing the three cancer cohorts, and the distribution of image cores across cancer cohorts and physical TMA arrays is summarized in Table S1. Model performance was evaluated using 10-fold cross-validation, with a fold-specific validation subset used during model fitting and a separate test set evaluated after training. To strike a balance between computational efficiency and information preservation, we resized the data to a more manageable size of 512 × 512 pixels. This resizing step retained the essential tissue features while significantly reducing the computational burden, enabling us to process and analyze the data more efficiently. For the additional physical-array-held-out and sample-level analyses, 1,208 image cores distributed across 10 physical TMA arrays were evaluated. These image-core counts are computational units and are distinct from the 568 clinical specimens summarized in Table 1. Their distribution across cancer cohorts and physical TMA arrays is summarized in Table S1.

### Data Preprocessing & Augmentation

Initially, we applied Otsu thresholding to identify and eliminate regions of the image lacking significant features. The original image (Figure 1A) was processed into an Otsu mask (Figure 1B) to filter out irrelevant areas and retain regions containing relevant information. Subsequently, we implemented a bounding box technique for cropping. A standard bounding box approach (Figure 1C) was followed by a smart bounding box technique (Figure 1D), which ensured precise cropping of the features of interest while minimizing the preserved area. To further enhance the dataset, we applied data augmentation techniques, including Mix-Up 16 (Figure 2A) and Cut-Mix 17 (Figure 2B), to improve data diversity, prevent overfitting, and boost model generalization.

### Model Architecture

We selected the EfficientNetB5 architecture 18 as the backbone network for our proposed method because of its strong performance in image classification tasks and favorable balance between accuracy and computational efficiency. The model was designed to process TMA images with dimensions of 512 × 512 pixels. Prior to feature extraction, several online data augmentation techniques were incorporated into the training pipeline, including random rotation, zooming, translation, contrast adjustment, brightness adjustment, and horizontal/vertical flipping, to improve model robustness and reduce overfitting. Subsequently, image pixel values were normalized to the range of [-1, 1] using a rescaling layer to ensure consistent input distribution.

As illustrated in Figure 3, the augmented and normalized images were fed into an EfficientNetB5 backbone initialized with Noisy Student pre-trained weights. The feature maps generated by the backbone network were then processed by a GlobalAveragePooling2D layer to aggregate spatial information into a compact feature representation. To further enhance generalization and mitigate overfitting, a Dropout layer with a rate of 0.2 was applied.

The resulting feature vector was subsequently passed through a fully connected Dense layer with 512 neurons to learn higher-level representations, followed by an additional Dropout layer with a rate of 0.2. Finally, a fully connected output layer with three neurons and a Softmax activation function was employed to generate the probabilities for the three target classes (Normal, Cancer, and Others), yielding the final classification prediction.

### Model Training & Evaluation

We trained the model for 80 epochs using the Adam optimizer 19, a learning rate of 1 × 10^-4^, and the categorical cross-entropy loss function for the multi-class classification problem. To dynamically adjust the training process, the learning rate was decreased by a factor of 0.1 at predetermined epochs, specifically at the 40th and 60th epochs. Gradient accumulation was implemented with a ratio of 16, enabling us to effectively simulate a batch size of 256 during training 20. To evaluate the performance of our proposed method, we utilized several key metrics, including F1-score, precision, recall, and accuracy. During 10-fold cross-validation, the fold-specific validation subset was used during model fitting, and a separate test set was evaluated after model training. Ten fold-specific EfficientNetB5 models were trained through this procedure. Ensemble results reported in the revised manuscript refer to the explicitly defined sample-level aggregation procedures described below.

### Robustness and Ensemble Analyses

Model performance was further examined under two complementary data-partitioning schemes. In the physical-array-held-out analysis, ten outer folds were defined at the physical TMA array level so that all cores from one physical TMA array were held out together for evaluation in each fold. The three-class classification task (Normal, Cancer, and Others) was retained throughout all analyses, with no further class merging during model training, prediction, or the additional robustness analyses. In the sample-level analysis, the 1,208-core dataset was partitioned at the sample level, allowing samples from the same source arrays to occur in different subsets. Predictions from ten fold-specific models were combined using an unweighted arithmetic mean of the three-class probability vectors for each sample, and the class with the highest mean probability was assigned as the final soft-ensemble prediction. As a sensitivity analysis, a hard plurality-vote ensemble was also evaluated using each model's maximum-probability class; when model votes were tied, the mean class-probability vector was used to resolve the tie. Overall classification performance under these additional analyses is summarized in Table S2.

## Results

### Cross-validation Performance

The performance of the EfficientNetB5-based model was evaluated using a 10-fold cross-validation framework. Classification performance was assessed using accuracy, precision, recall, and F1-score metrics. Across the validation folds, mean accuracy, precision, recall, and F1-score were 85.93%, 82.30%, 84.53%, and 82.78%, respectively (Table 2). The test set was evaluated using each fold-specific model; across the ten models, mean accuracy, precision, recall, and F1-score were 80.08%, 74.98%, 76.45%, and 75.13%, respectively (Table 3).

### Cancer Detection Performance

To further evaluate Cancer-class detection, precision, recall, and F1-score were calculated for the Cancer class (Table 4). Across the cross-validation validation folds, Cancer precision was 100%, with mean recall of 87.10% and mean F1-score of 93.06%. Across the separate test-set evaluations by the ten fold-specific models, mean Cancer precision, recall, and F1-score were 99.94%, 86.59%, and 92.78%, respectively. A Cancer precision of 100.00% indicates that no Normal or Others samples were observed among samples predicted as Cancer; it does not indicate that all Cancer samples were detected.

### Sample-Level Ensemble Performance

In the sample-level analysis, ten fold-specific models were combined using the explicitly defined unweighted mean-probability rule. On the 246-core test subset, this soft ensemble achieved 87.80% accuracy, 76.20% macro precision, 80.88% macro recall, and 78.18% macro F1-score; Cancer precision was 100.00% and Cancer sensitivity was 91.62%. The hard plurality-vote sensitivity analysis, with mean-probability tie-breaking, achieved 86.99% accuracy, 74.53% macro precision, 78.93% macro recall, and 76.38% macro F1-score. The two aggregation rules differed for only 2 of 246 predictions (Table S2).

## Discussion

The results of this study demonstrate the feasibility of applying EfficientNetB5 to automated TMA image classification. Across the 10 cross-validation validation folds, mean accuracy and F1-score were 85.93% and 82.78%, respectively. Across the separate test-set evaluations by the fold-specific models, mean accuracy and F1-score were 80.08% and 75.13%, respectively. In the sample-level analysis, the explicitly defined 10-model mean-probability ensemble achieved 87.80% accuracy and 78.18% macro F1-score on the 246-core test subset, while the hard plurality-vote sensitivity analysis achieved 86.99% accuracy and 76.38% macro F1-score. Cancer precision also requires careful interpretation: a precision of 100% indicates that no Normal or Others samples were observed among predictions assigned to the Cancer class; it does not mean that every Cancer sample was detected. Compared with former methods, our approach offers several advantages:

- It employs a deep learning method to classify TMA images into Normal, Cancer, and Others.
- We utilize TMA array images as input, eliminating the need for time-consuming and expert labor-intensive handcrafted features.
- The framework achieved high Cancer precision in the reported evaluations, indicating few false-positive Cancer predictions.

For example, ImageMiner 21 is one of the earliest software platforms for analyzing TMA images with the combination of image processing methods for tasks such as registration, segmentation, feature extraction, and tumor classification. However, it does not provide classification or evaluation of the risks or potential development of a cancer disease. Anant Madabhushi et al. 22 have presented a review of TMA analysis, particularly focusing on research in tissue classification and cancer grading for predictions. Other methods, like those by Veta et al. 23, utilize handcrafted features, including nuclear shape and texture, for prognostic purposes. Similarly, Basavanhally et al. 24 use nuclear architecture, shape, and texture to estimate cancer grading. In contrast, Bychkov et al. 25 employ deep learning methods to predict survival and cancer grading. However, these methods primarily target survival and cancer grading but do not provide disease predictions for early treatment. On the other hand, Yan et al. 26 utilize deep features and employ a combination of machine learning and deep learning methods to achieve an impressive error rate of 23.28% in estimating cancer, negative (normal), and other conditions. Our approach stands out by providing a comprehensive solution for TMA image analysis that not only achieves high performance but also addresses some of the limitations of previous methods. However, our study also has important limitations. First, all inputs were resized to 512 × 512 pixels, which may remove fine histopathologic detail. Second, although each cancer cohort included corresponding adjacent normal and tumor tissues, aggregate performance alone should not be interpreted as evidence of uniform generalizability across cancer types, acquisition conditions, or unseen datasets. Because the cohorts were assembled from distinct TMA sources, source-associated differences in staining, tissue preparation, or image acquisition may remain correlated with class labels and may partly contribute to the observed aggregate performance. Future work should prioritize source-balanced evaluation, independent multicenter cohorts, higher-resolution inputs, and model-interpretability analyses to determine whether performance generalizes across TMA sources and cancer populations.

## Conclusion

We developed an EfficientNetB5-based deep learning framework for automated tissue microarray image analysis and Cancer-class detection. Across cross-validation and test-set evaluations, the model showed useful aggregate classification performance and high Cancer precision; however, precision should be interpreted as the absence of observed false-positive Normal/Others samples among Cancer predictions rather than complete Cancer detection. These results support the feasibility of deep learning-assisted TMA classification while indicating that aggregate metrics alone are insufficient to establish uniform robustness or generalizability across TMA sources. Further validation using source-balanced evaluation and independent multicenter cohorts is required before clinical application.

## Supplementary Material

Supplementary tables.

## Figures and Tables

**Figure 1:**
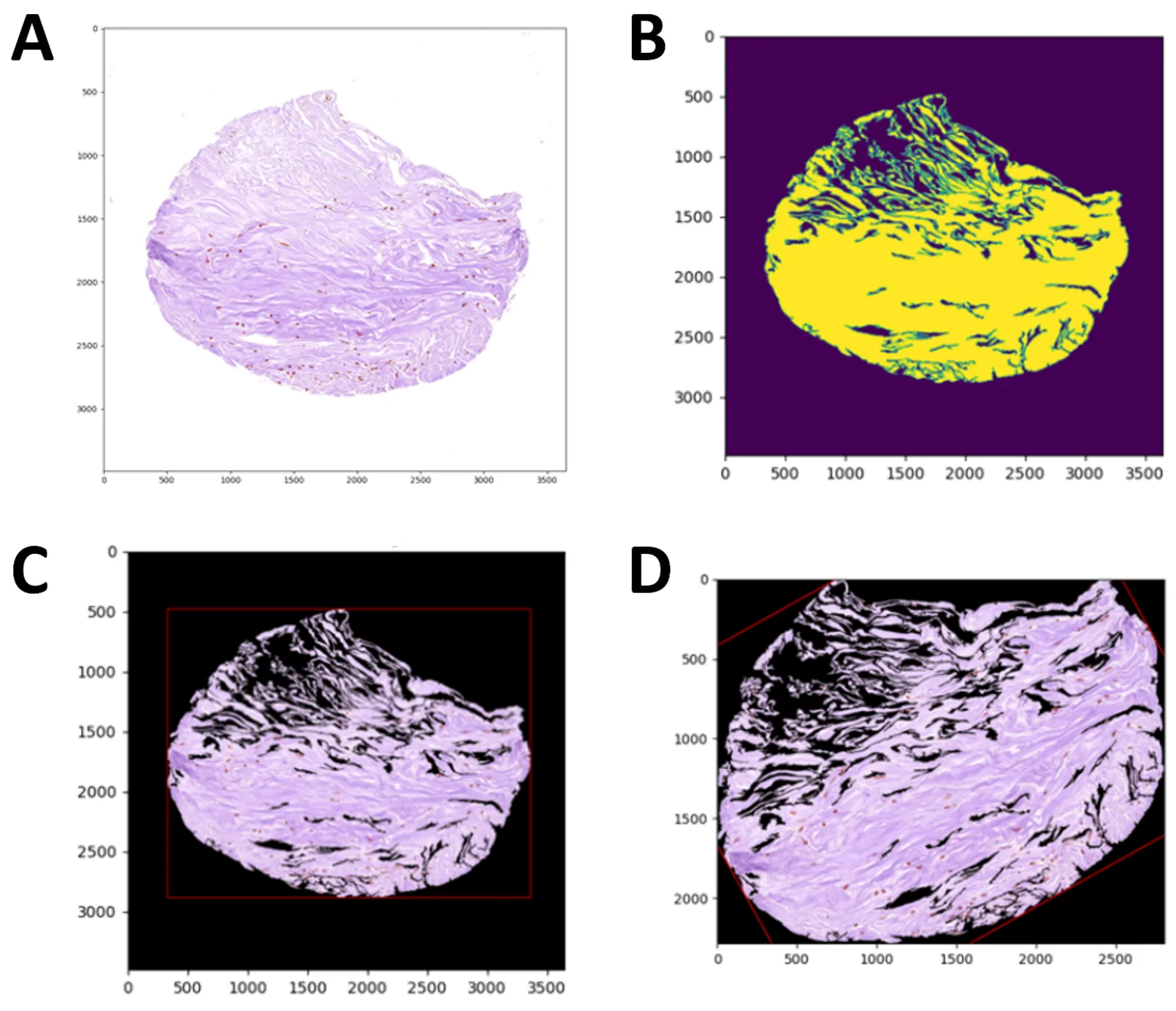
** Illustration of data preprocessing step.** (A) Original image. (B) Otsu mask. (C) Normal bounding box. (D) Smart bounding box.

**Figure 2:**
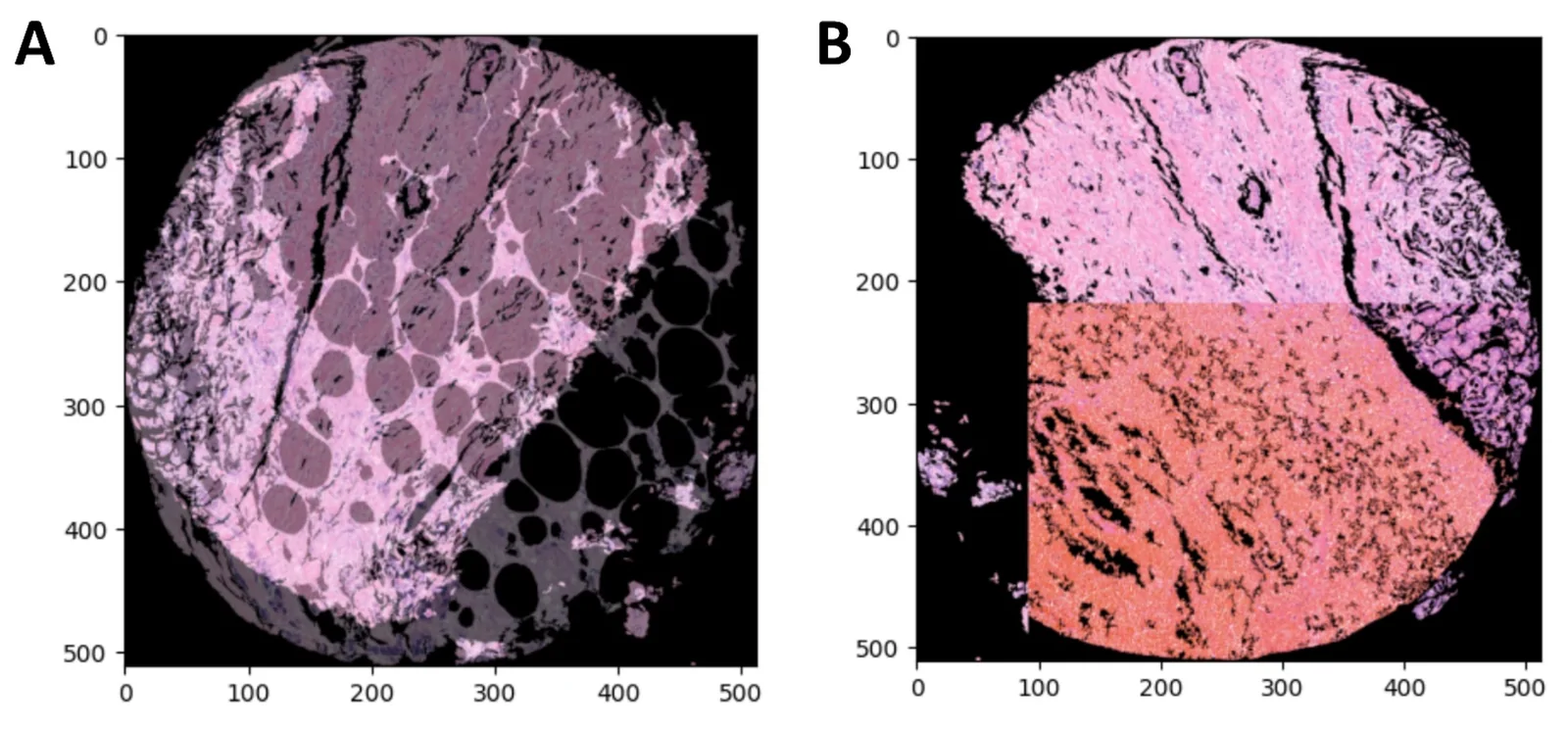
** Illustration of data augmentation techniques.** (A) Mix-Up. (B) Cut-Mix.

**Figure 3:**
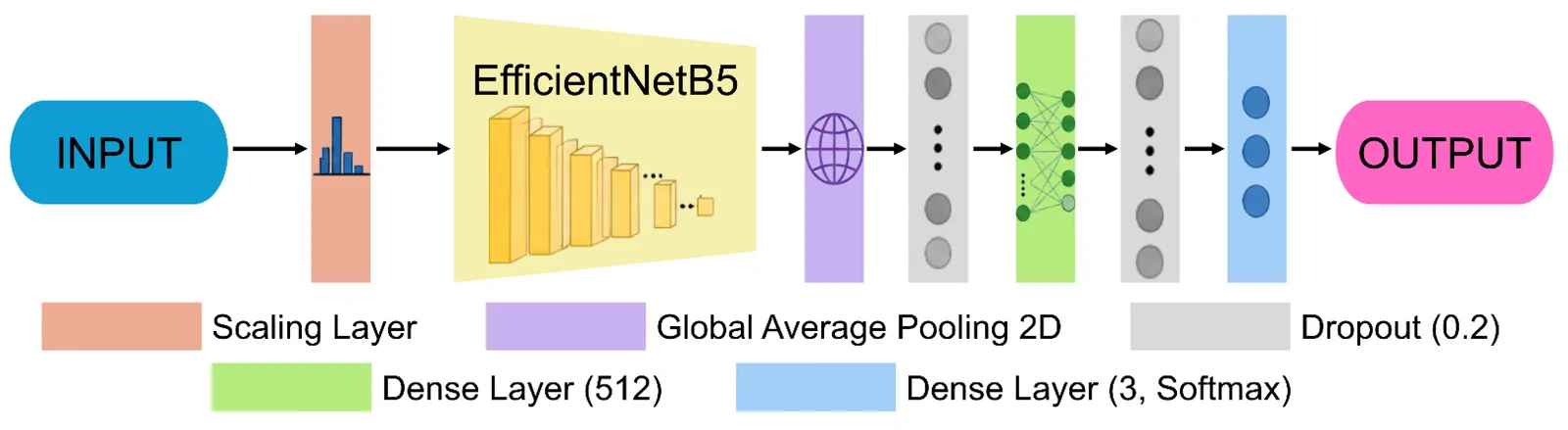
Overall model architecture.

**Table 1:** Composition of the clinical tissue microarray cohorts.

Cancer type	Samples
Breast cancer	309
Gastric cancer	110
Upper Tract Urothelial Carcinoma (UTUC)	149
Total	**568**

**Table 2:** Evaluation metrics across the 10 cross-validation validation folds.

Validation folds				
Fold	Accuracy (%)	Precision (%)	Recall (%)	F1-score (%)
1	89.29	85.71	88.59	86.83
2	88.57	85.24	89.07	86.56
3	87.86	84.43	87.83	85.61
4	86.43	83.17	87.83	84.27
5	84.89	80.66	84.65	81.69
6	81.29	76.75	77.70	76.77
7	83.45	81.70	80.00	79.95
8	87.05	82.00	82.81	82.30
9	87.05	83.07	82.44	82.53
10	83.45	80.31	84.41	81.32
Average	85.93	82.30	84.53	82.78

Note: Accuracy represents the overall proportion of correctly classified samples. Precision, recall, and F1-score are macro-averaged across the three target classes (Normal, Cancer, and Others). Average values represent the arithmetic mean across the ten cross-validation folds.

**Table 3:** Evaluation metrics on the test set across the ten fold-specific models.

Test set				
Fold	Accuracy (%)	Precision (%)	Recall (%)	F1-score (%)
1	78.71	74.09	76.20	74.07
2	78.43	72.63	73.68	72.77
3	79.27	74.09	75.46	74.35
4	80.67	76.37	79.16	76.75
5	81.79	77.06	79.21	77.66
6	80.11	74.36	74.65	74.19
7	80.67	75.67	77.03	76.01
8	80.95	76.08	78.18	76.59
9	79.27	73.04	71.91	71.94
10	80.95	76.41	79.06	76.92
Average	80.08	74.98	76.45	75.13

Note: Accuracy represents the overall proportion of correctly classified samples. Precision, recall, and F1-score are macro-averaged across the three target classes (Normal, Cancer, and Others). Average values represent the arithmetic mean across the ten fold-specific test-set evaluations.

**Table 4:** Cancer-class performance across the cross-validation validation folds and separate test-set evaluations.

	Validation folds†			Test set‡		
Fold	Precision (%)	Recall (%)	F1-score (%)	Precision (%)	Recall (%)	F1-score (%)
1	100.00	91.30	95.45	100.00	86.93	93.01
2	100.00	88.41	93.85	100.00	86.36	92.68
3	100.00	86.96	93.02	100.00	85.80	92.35
4	100.00	82.61	90.48	100.00	85.80	92.35
5	100.00	88.41	93.85	100.00	86.93	93.01
6	100.00	84.06	91.34	100.00	86.93	93.01
7	100.00	82.61	90.48	99.35	86.36	92.40
8	100.00	92.75	96.24	100.00	86.36	92.68
9	100.00	92.75	96.24	100.00	88.07	93.66
10	100.00	81.16	89.60	100.00	86.36	92.68
Average	100.00	87.10	93.06	99.94	86.59	92.78

Note: Precision, recall, and F1-score are reported specifically for the Cancer class. **†** Validation-fold performance was calculated on the held-out fold in each cross-validation run.**‡** Test-set performance was calculated on the separate test set using each of the ten fold-specific models.

## Data Availability

The data that support the findings of this study are available from the corresponding author upon reasonable request.

## Abbreviations

TMA: Tissue microarray
H&E: hematoxylin and eosin
UTUC: upper tract urothelial carcinoma
IRB: institutional review board
CNN: convolutional neural network
