# Supplementary material for: EfficientNetB5-Based Deep Learning for Automated Cancer Detection in Tissue Microarray Images
Source: J Cancer. 2026 Sep 24;17(10):1821–8. doi: 10.7150/jca.139302 (PMC13617411; doi:10.7150/jca.139302)
Supplement: Supplementary file 1 [file jcav17p1821s1.pdf]

Supplementary Tables

Table S1 Distribution of image cores across cancer cohorts and physical TMA arrays.

Cancer type	Physical TMA arrays	No. of arrays	Image cores, n
**Breast cancer**	Breast_1-1, Breast_1-2, Breast_1-3, Breast_1-4	4	454
**Upper Tract Urothelial Carcinoma (UTUC)**	UTUC_1M01, UTUC_1M02, UTUC_1M03	3	439
**Gastric cancer**	Gastric_1M03, Gastric_1M04, Gastric_1M06	3	315
**Total**	—	**10**	**1,208**

Table S2 Classification performance in the additional analyses.

Evaluation scheme†	Accuracy (%)	Precision (%)	Recall (%)	F1-score (%)
**Physical-array-held-out analysis‡**	82.53	65.70	68.05	66.54
**Sample-level soft mean-probability ensemble§**	87.80	76.20	80.88	78.18
**Sample-level hard plurality-vote ensemble§**	86.99	74.53	78.93	76.38

† **Precision, recall, and F1-score are macro-averaged across the three target classes (Normal,** Cancer**, and** Others**).**

‡ **The physical-array-held-out values are pooled out-of-fold results across 1,208 image cores.**

§ **The sample-level ensemble values are based on the 246-core test subset. The soft ensemble used the unweighted arithmetic mean of the class-probability vectors from ten fold-specific models, whereas the hard ensemble used plurality voting with mean-probability tie-breaking.**
